# Exploring the Intersection of Hegemonic Masculinity, Sexuality, and Addiction in Men: A Qualitative Study

**DOI:** 10.3390/healthcare13010005

**Published:** 2024-12-24

**Authors:** Julio A. Camacho-Ruiz, Carmen M. Galvez-Sánchez, Federica Galli, Rosa M. Limiñana-Gras

**Affiliations:** 1 Foundation Project Man Jaén, 23002 Jaén, Spain; julioangel.camachor@um.es; 2 Department of Personality, Evaluation and Psychological Treatment, Faculty of Psychology and Speech Therapy, University of Murcia, Building 31, 30100 Murcia, Spain; liminana@um.es; 3 Regional International Campus of Excellence (CEIR) Mare Nostrum Campus (CMN), 30100 Murcia, Spain; 4 Department of Dynamic and Clinical Psychology, and Health Studies, Faculty of Medicine and Psychology, SAPIENZA University of Rome, 00185 Rome, Italy; f.galli@uniroma1.it; 5 Assisted Reproduction Unit, QuironSalud Murcia Medical Center, 30008 Murcia, Spain

**Keywords:** sexuality, hegemonic masculinity, addiction, men, self-esteem

## Abstract

**Background/Objectives:** In our society, as well as in many other parts of the world, sexuality is shaped through gender-differentiated socialization. This process compels individuals to align their desires, behaviors, emotions, and thoughts with the expectations of normative sexuality, especially hegemonic heterosexuality. The primary objective of this current research was to examine the influence of hegemonic masculinity on the sexuality of men struggling with addiction. **Method:** This study employed a qualitative approach, specifically using conventional content analysis. To ensure research quality and transparency, the study adhered to the Standards for Reporting Qualitative Research (SRQR) and the Consolidated Criteria for Reporting Qualitative Studies (COREQ). Fourteen participants from a therapeutic community for addiction treatment in Spain were selected through purposive sampling. Data collection involved semi-structured interviews, supplemented by participant observation. The sample size was determined based on the principle of data saturation. **Results:** The findings reveal that factors such as gender-differentiated socialization, particularly the values associated with hegemonic masculinity (e.g., the pressure to maintain constant sexual availability and sexual initiative), alongside excessive engagement with prostitution and pornography, are closely linked to substance abuse, the development of addictions, and the emergence of sexual dysfunctions in men. The study also highlights the role of challenges related to oppression rooted in heteronormativity—understood as the imposition of rigid norms governing sexual orientation and behavior—in shaping sexual problems and in the origin and maintenance of addictions in men. **Conclusions:** It is essential to implement an intervention strategy that promotes egalitarian masculinities within the treatment of men with addiction issues, emphasizing a biopsychosocial approach to sexuality that integrates a gender perspective. Additionally, it is crucial to incorporate gender-sensitive interventions into rehabilitation programs for both men and women, ensuring a comprehensive understanding of each group’s specific needs and fostering the development of healthy, equitable relationships in the context of addiction treatment.

## 1. Introduction

### 1.1. Sexuality as a Multidimensional Phenomenon Analyzed from a Gender Perspective

In contemporary society, sexuality remains both a prominent and taboo subject. Although it is pervasive in cinema, everyday conversations, the internet, and music, meaningful discussions and critical analyses are often constrained by societal restrictions and taboos, fostering a double standard that negatively impacts many individuals. Addiction issues are frequently associated with challenges in conceptualizing and experiencing sexuality, particularly among those undergoing treatment [1]. Furthermore, educational processes and socialization perpetuate gender-based stereotypes related to sexuality (e.g., men are expected to initiate sexual activity, maintain constant sexual availability, and view promiscuity as a masculine virtue). These stereotypes profoundly influence the identities of individuals in rehabilitation, adversely affecting their interpersonal relationships, self-concept, and self-esteem.

It is important to highlight that men are seldom the focus of social interventions informed by a gender perspective [2], and even less so when it comes to applying this perspective to issues related to sexuality. This dynamic stems from an androcentric worldview in which the white, Catholic, heterosexual, middle-class, middle-aged man has been positioned as the reference framework for interventions aimed at other groups, rather than as a subject of intervention himself [3].

Integrating the concept of self-esteem into the development of sexuality, and vice versa, is essential, as negative sexual experiences faced by many men in rehabilitation programs have significantly contributed to the onset and persistence of their addiction issues. The interplay between diminished self-esteem and negative sexual experiences underscores how these factors interact and exacerbate vulnerability to problematic substance use.

In Marcela Lagarde’s [4] definition of self-esteem, a subjective dimension is proposed, rooted in emotions, desires, events, and experiences. When incorporating this dimension into the construction of self-esteem in many men, it is crucial to examine how these concepts have been shaped through socialization processes, as well as the relationship between sexuality and self-esteem. According to Ranea [5], the concept of virility, constructed within male peer groups, plays a significant role in shaping male desire. For men, as bell hooks [1] argues, this desire is more influenced by quantity than quality. Consequently, many men perceive their self-esteem as closely tied to the number of heterosexual encounters they have throughout their lives. Furthermore, these experiences must be validated by their peer groups, which are primarily responsible for conferring prestige on the “conquering” man and labeling him as successful and virile. Thus, particularly in the context of sexuality, men require external validation of their self-esteem, which is reinforced by their peers [1]. This is significant because, as previously noted, men benefit from patriarchy through hegemonic masculinity. This form of masculinity is defined by the domination of public spaces, the subordination of women to the private sphere, and the acquisition of prestige, status, and power. In this unequal society, men utilize a highly effective tool: violence. Violence is fundamental to asserting domination, not only over women but also over other men [6]. Thus, hegemonic masculinity functions as a mechanism of domination across social, cultural, political, and economic domains, sustaining violence as a means of controlling subordinated individuals [5].

Building on Lagarde’s [4] definition, individuals construct their self-esteem based on acquired experiences, many of which stem from interactions within peer groups and positive emotions associated with proactive behaviors. These behaviors sometimes link individuals to success, while at other times, they lead to frustration due to unfulfilled desires and, occasionally, feelings of shame and fear for failing to meet their peers’ expectations of being the virile men they are expected to be, as well as the societal stereotypes imposed on them. It can be concluded that the concept of self-esteem for many men is inseparable from the heteronormativity imposed as a core value of hegemonic or dominant masculinity within the prevailing patriarchal system.

For these reasons, in our society and many other parts of the world, sexuality is constructed through gender-differentiated socialization. This process leads individuals to align their desires, behaviors, emotions, and thoughts with what is dictated by normative sexuality, particularly hegemonic heterosexuality. This conception of sexuality is primarily shaped by cultural, historical, and religious factors [5]. Throughout our lives and during our socialization process, we are often confronted with the notion that men enter relationships primarily motivated by the pursuit of sex, while women seek love. Moreover, there has been a strong emphasis on educating men with the idea that emotions must necessarily be fulfilled through sexual commitment [1]. All of this has a negative impact on the sexuality of both genders.

### 1.2. Hegemonic or Dominant Masculinity: Its Gender Norms and Impact on Sexuality

It is crucial to address two gender norms or mandates that underlie the sexual education men receive through gender-differentiated socialization, influenced by hegemonic masculinity. The first norm pertains to the expectation that men must take the initiative, meaning they are expected to be active agents in seeking sexual encounters, while women are positioned as passive objects. Consequently, this norm designates men as the possessors of desire [1]. This gender norm not only creates inequalities for women, who are stigmatized if they dare to openly embrace behavior typically associated with men, such as seeking promiscuous sexual encounters, but it also penalizes men who either choose not to adhere to these norms or lack the social skills to secure multiple sexual encounters. The second gender norm, imposed by patriarchy and continuously reinforced by peer groups (similar to the reinforcement of the first norm), asserts that men must always be available for sex. It is often considered strange, or even offensive, to suggest that a man might not want to engage in sexual activities proposed openly by women.

There are significant social punishments or sanctions for men who fail to conform to the sexual mandates imposed by patriarchy, such as perpetual sexual availability, initiative, and penetration as symbols of complete sexual relations. Consequently, these social punishments experienced by men result in feelings of shame associated with male impotence and/or failure to adhere to the core values of heteronormativity. In many cases, these oppressive behaviors dictated by heteronormativity contribute to the development of addictive behaviors in numerous men. This highlights the relevance of analyzing the potential influence of gender norms and stereotypes associated with hegemonic masculinity on addiction.

In the domain of sexuality, men enjoy several important privileges [7], including the following:

(A) It is highly unlikely for a man to be a victim of rape. A man can walk peacefully at night without feeling the need to cross the street when another man approaches, nor does he need to make a phone call to someone he knows while walking alone [7].

(B) Desire and initiative: Women’s desire is often associated with promiscuity; for a woman, promiscuity is synonymous with being a “bad” woman. There exists a deeply ingrained double standard in many societies, where male sexuality is perceived differently from female sexuality [7]. While sexual promiscuity in men is often tolerated or even celebrated as a symbol of prestige and power, the same behavior in women is commonly stigmatized.

(C) A man is not typically stigmatized for engaging in prostitution. In the context of prostitution, men generally do not face significant stigmatization for participating in this activity, except when they engage in prostitution with other men. This exception is particularly noticeable among men from vulnerable socioeconomic backgrounds, where power dynamics also influence relationships [7]. In society, men who engage in prostitution with other men encounter considerable stigma, primarily because these practices deviate from established heteronormative standards and the ideal of hegemonic masculinity. These men experience a double stigmatization: on one hand, the general social judgment of prostitution, and on the other, the specific disapproval arising from dominant masculine ideals, which not only reject sexual expressions that deviate from heterosexuality but also penalize dynamics of vulnerability and economic dependence.

(D) Penetration as a marker of a complete sexual relationship: A thorough examination of pornographic videos available on various websites is sufficient to illustrate this point. In the vast majority of these videos, sexual intercourse involves the penetration of the woman, who is frequently depicted as submissive and awaiting penetration [7]. This representation reinforces the normative expectation of heterosexuality and positions penetration as the exclusive means of achieving pleasurable sexual experiences. In doing so, it creates an ideology that views the phallus, or male genitals, as the central organizing element of the social world—an idea known as phallocentrism.

Moreover, there are various costs associated with men’s sexuality, stemming from experiences shaped by the mandates of hegemonic masculinity [7], such as those listed below:

(A) Maintaining initiative, desire, and managing frustration in the face of rejection: A significant cost is the pressure on men to consistently take the sexual initiative and display a high level of desire. This expectation can lead to several issues, both for the men who internalize this norm and for their partners.

(B) The expectation of constant availability for sex: The belief that men must always be ready and willing to engage in sexual activity can create significant psychological pressure. This pressure may lead to feelings of anxiety, stress, and excessive self-demand, particularly when men perceive that they are failing to meet this expectation. Such feelings can negatively affect their self-esteem, emotional well-being, and sexuality.

(C) Handling rejection: Hegemonic masculinity also dictates that men must be able to accept rejection calmly and without displaying vulnerability. However, the reality is that sexual rejection can be challenging for many individuals, regardless of gender. Men who internalize the belief that they must always succeed in their sexual encounters may experience intense feelings of frustration, shame, or even anger when confronted with rejection [1]. This can contribute to an unequal power dynamic in sexual relationships, where women’s needs and desires are subordinated to those of men.

(D) Social pressure of having multiple sexual partners: Moreover, the need of engaging in numerous sexual encounters [1] can lead to risky behaviors, such as unprotected sex or sex with multiple partners, thereby increasing the likelihood of contracting sexually transmitted infections (STIs) or experiencing unintended pregnancies. These behaviors may also negatively impact the onset and maintenance of addictions, as substances may be used as a means of socialization or to gain the confidence necessary to participate in these multiple sexual encounters. All of these negative consequences may arise from the misconception that engaging in numerous sexual encounters is a marker of virility and status among men.

(E) Emotional connections: On a deeper level, the need on engaging in numerous sexual encounters may obstruct the establishment of meaningful emotional connections with partners. This can lead to feelings of emotional emptiness and dissatisfaction in sexual relationships, as the focus shifts more toward quantity rather than the quality of intimate experiences [1]. In this context, addictions may also emerge as a means to fulfill these emotional needs and fill that void.

These findings underscore the importance of incorporating a gender perspective in interventions for men with addiction issues. A gender-sensitive approach not only helps to address inequalities related to the experiences and development of sexuality between men and women but also challenges the norms imposed by hegemonic and dominant masculinity, contributing to the formation of egalitarian masculinities.

### 1.3. Heteronormativity as the Basis for the Development of Sexuality in Hegemonic Masculinity

Drawing on Judith Butler [8], the male supremacist paradigm—which we would also specify as white, as bell hooks [1] aptly emphasizes—highlights a heterosexuality that applies to both men and women, thereby establishing the heterosexual imperative as an institutional norm. This imperative presupposes that heterosexual relationships are natural. Consequently, the patriarchal system mandates the expression of sexuality through desire between men and women. The patriarchy and its heterosexual imperative are intrinsically linked to sexual potency, which is transmitted from fathers to sons, thus transforming it into a form of symbolic and social capital. This way of portraying sexuality not only repressively rejects sexual relationships that fall outside the values and norms of heteronormativity but also fosters a homophobic sentiment that dismisses alternative expressions of sexuality [8].

Hegemonic or dominant masculinity, rooted in patriarchal paradigms, imposes a restrictive view of sexuality that privileges heterosexual behaviors and penalizes those that deviate from heteronormative expectations [8]. This exclusionary approach not only marginalizes individuals who do not conform to traditional and dominant gender norms but also imposes significant sexual costs on both individuals and entire communities.

It is essential to understand the relationship between dominant or hegemonic masculinity and heteronormativity. Sexuality develops and assumes significant relevance within gender-differentiated socialization, as it is intrinsically linked to gender norms and roles for both women and men. From birth, it is presumed that all individuals are heterosexual, and those who deviate from this norm often experience societal rejection. Heteronormative sex is the form that affirms masculinity and virility in men, particularly within peer groups [5]. Another important concept to consider is the penis as the focal point of sexual and power relations, which is evident in much of the pornography consumed by men.

The significance of analyzing sexuality and heteronormativity within the context of addictive behaviors is paramount. Heteronormativity influences adolescent development and plays a crucial role in shaping relationships within peer groups. To conduct a comprehensive analysis of the topic, it is essential to discuss the concept of heteronormativity. According to Welzer-Lang [9], it is the system that currently exerts male dominance over women and other men who do not conform to certain ideals that sustain its hegemony [9]. This phenomenon not only separates women and men physiologically but also operates between genders.

This manner of experiencing sexuality serves as a tool of discrimination and oppression, not only against homosexual individuals but also against any alternative forms of engaging in sexual relationships that deviate from established norms. On some occasions, homophobia arises from the fear that allowing boys to express emotions may influence their potential sexual orientation toward homosexuality [1]. Every day, many children face psychological aggression for expressing feelings that may be categorized as feminine. Patriarchal paradigms regarding sexual behaviors render only those practiced by heterosexual men as desirable [1]. In some instances, within contexts involving drug use, men may simultaneously engage in oppressive sexual relationships—such as prostitution, sexual dysfunction, abuse, and sexual violence—or relationships that deviate from established heteronormativity, leading to existential and emotional crises that often contribute to substance abuse and the onset of addiction [9].

Furthermore, non-heteronormative sexual experiences and experimentation are often linked to the exacerbation of substance abuse or serve as a foundation for the development of addiction issues. In addiction treatment settings, it is not uncommon to encounter men who have silently suffered as a result of these types of relationships. Therefore, it is essential to create spaces where these matters can be addressed with the highest level of empathy.

### 1.4. Prostitution and Pornography: The New Pedagogy of Sexuality for Hegemonic and Heteronormative Masculinity

Another consequence experienced by some men, which bears many similarities to addictions to alcohol or drugs, is the addiction to pornography and sex [1]. For many men, sex serves as a mechanism of self-soothing that helps to fill an existential void. It becomes a means of alleviating their pain, resembling the behaviors of individuals with addiction issues. The addicted individual often carries significant emotional distress. For many men, sexuality assumes this addictive dimension [1]. Given their limited free time each day, it is often impossible for men to engage in multiple sexual relationships; thus, pornography provides an accessible avenue for sex addicts to satisfy this compulsion. Today, pornography can be accessed at any time of the day, thanks to immediate internet availability. Moreover, for men, this approach to experiencing sexuality—through pornography consumption—provides a sense of sexual power, which can often result in directing frustration and resentment toward women perceived as genuinely possessing sexual power. This dynamic is particularly evident in the consumption of prostitution, where many men attempt to reassert control and reclaim power over women [1].

Another normalized practice within patriarchal sexuality is prostitution. Prostitution reinforces the notion for men that certain individuals lack intrinsic value and are merely tools to achieve their own objectives [10]. This type of relationship prevents men from treating women with empathy, equality, and dignity, thereby perpetuating a sense of superiority over them. Assigning a monetary value to a woman’s body dehumanizes and objectifies her, reducing her to an object and aligning her with a system comparable to slavery. This type of relationship distances men from treating women with empathy, equality, and dignity, thereby maintaining a position of superiority over them. Assigning a price to a woman’s body dehumanizes her, treating her like an object and aligning her with a system akin to slavery.

In addition, prostitution is an increasingly common practice that sometimes serves as a rite of initiation into heterosexual relationships for many young people, often reinforcing virility through group dynamics [5]. These initiations are frequently accompanied by excessive consumption of alcohol and other substances. In fact, most venues where prostitution occurs increasingly resemble entertainment spaces that sell alcoholic beverages, such as pubs and nightclubs. Additionally, the excessive consumption of alcohol and other substances often acts as a way to lose their inhibitions, facilitating engagement in prostitution.

### 1.5. Theoretical Framework

This research is grounded in feminist theory and gender studies, which provide a critical framework for analyzing the social constructions of masculinity, power dynamics, and the intersection of gender with other social categories such as ethnicity and socioeconomic status. By integrating feminist theory with social constructionism and critical masculinity studies, the research aims to explore the influence of hegemonic masculinity on the sexuality of men with addiction, considering the broader social, cultural, and psychological factors involved.

Additionally, the study incorporates the Biopsychosocial Model to examine gender-specific patterns of addiction, addressing biological, psychological, and social dimensions and their impact on substance use, relationships, and gender dynamics [11]. In the context of addiction, the model facilitates an analysis of biological aspects (e.g., the damage caused by drugs and irresponsible sexuality to the health of those undergoing treatment), psychological aspects (e.g., personality traits and poor emotional regulation, such as jealousy or the mismanagement of unsatisfactory or oppressive sexual relationships, which may become risk factors for substance use, potential shifts in values, biases regarding masculinity, and attitudes towards drug use and the emotional-sexual domain), and social-community-peer group dynamics (e.g., issues related to social pressure and its impact within peer groups, once individuals have undergone rehabilitation, as well as gender-based or sexual violence, and codependent relationships) among the users involved.

It is precisely its holistic and comprehensive perspective on the issue that lends this model greater strength and effectiveness. This is why it is highly relevant for gender-focused interventions, particularly when gender is understood as a sociocultural construct [12]. According to Benería [13], the concept of gender is shaped by an amalgamation of thoughts, behaviors, values, desires, emotions, roles, and personal traits, which are differentially ascribed to men and women through a social construction process. Therefore, it is clear that this model is particularly well-suited for gender-sensitive interventions. Specifically, this research has adhered to the Ecological Feminist Model [14] and the gender perspective in health [15,16].

#### Ecological Feminist Model and Gender Perspective in Health

The Ecological Feminist Model advocates for an intervention that considers the need to address social inequalities, gender-based violence, and changes across all levels and structures of society. It shares with the Biopsychosocial Model the focus on individual, social, group, and community characteristics, both of which are frameworks proposed by the WHO for addiction interventions [15,17]. What distinctly differentiates this approach, particularly in its application to men, is its foundation in an intersectional perspective. This means it incorporates political and cultural factors, requiring men’s active involvement in all efforts aimed at promoting equality and eliminating violence against women. It seeks to transform men into critical agents who challenge the powers and public administrations responsible for creating social policies that eradicate inequality [14].

In line with the proposals of this model, specific research lines would be identified. These would focus on highlighting the power imbalances that persist, a heteronormative and dominant masculinity that dictates specific gender roles regarding the expression of male sexuality, the pornification of sexuality, and the violence in media that sexualizes and commodifies the female body. Additionally, poverty would be addressed as a mechanism that limits men’s access to material resources, particularly educational ones.

Furthermore, on a subjective level, it is crucial to analyze the role of peer groups in differentiated socialization processes, adherence to gender norms, and the internalization of stereotypes and roles conveyed by various socialization agents, in addition to the peer group itself [14].

The determinants of health linked to gender encompass the norms, roles, and social expectations that shape the risks and vulnerabilities individuals face based on their gender, as well as their ability to access protection against these health risks. Gender also influences decisions regarding the adoption of healthy habits and overall health, while shaping healthcare systems’ responses to individuals based on their gender. These gender dynamics are key factors contributing to health inequities, as they shape the conditions under which individuals experience health and illness, as well as their access to medical services [15].

Integrating a gender perspective into health studies entails more than simply disaggregating data by sex, although this practice represents a crucial initial step. A comprehensive approach accounts for both sex and gender while challenging the assumption that all individuals—regardless of gender identity (men, women, transgender, transsexual, or non-binary identities)—experience health issues uniformly or respond similarly to treatments. In reality, experiences of health and related challenges vary significantly, shaped by power structures and gender norms that influence individuals’ access to healthcare, emotional well-being, and capacity to seek help [16].

For that reason, the gender perspective in health is also incorporated into this research as a central element of its conceptual framework.

### 1.6. Objectives

The general objective was to explore the impact of hegemonic masculinity on the sexuality of men facing addiction from a qualitative perspective. The specific objectives were as follows: (1) to analyze the gender norms or mandates imposed by hegemonic masculinity and their impact on the sexuality of men with addiction; (2) to explore the consumption of pornography and prostitution, along with their associated costs, in men with addiction; and (3) to analyze heteronormativity as a mechanism of oppression for certain men undergoing addiction treatment. The research question was: How does hegemonic masculinity impact the sexuality of men facing addiction?

## 2. Materials and Methods

### 2.1. Methodological Approach

The methodology selected for analyzing the chosen topic is qualitative, with a specific focus on content analysis, considered the most suitable approach due to its ability to explore the reproduction of gender inequalities [18].

A qualitative methodology enables the examination of the shifting dynamics between men and women, highlighting key elements and providing a framework for interpreting and integrating participants’ viewpoints [18]. This method encourages open expression and allows for the exploration of subjectivity, without the limitations imposed by other approaches.

To maintain methodological rigor, this research follows the Standards for Reporting Qualitative Research (SRQR) [19] as well as the Consolidated Criteria for Reporting Qualitative Studies (COREQ) [20]. These guidelines play a crucial role in ensuring transparency and strengthening the credibility of the study’s outcomes.

### 2.2. Participants: Inclusion Criteria and Characteristics

The research project was conducted with fourteen individuals facing addiction challenges who are currently engaged in rehabilitation programs at the Foundation Project Man of Jaén, located in the provincial capital and operating within a provincial framework. The primary demographic profile consists predominantly of white males, leading to a notable underrepresentation of individuals from other nationalities or ethnic backgrounds. Importantly, even though the organization is non-confessional and non-partisan, the majority of participants identify as either non-believers or Catholics. There are socioeconomic disparities among the participants, with a significant portion coming from families with a middle socioeconomic status. However, there are also families with a medium-high socioeconomic profile, often indicating previous attendance at private rehabilitation centers, suggesting their preference for private institutions over those in the third sector, in addition to individuals from lower socioeconomic backgrounds.

Fourteen male participants were selected for this research using purposive sampling (see Table 1: Participant Characteristics). The determination of sample size was informed by the principle of information saturation [21]. Recruitment persisted until the point of redundancy in the information was reached, ensuring sufficient data for potential study replication and making further coding efforts impractical [22]. The inclusion criteria consisted of male gender, a diagnosed addiction issue, and active participation in the therapeutic community at Foundation Project Man, either through rehabilitation programs or outpatient treatment initiatives. Exclusion criteria included individuals with less than seven months of treatment in any of the specified programs and those without severe mental disorders. All eligible participants were invited to take part in the study by the administration of the center.

No prior relationship was established between the interviewer and potential participants before the commencement of the study. Participants were informed about the researcher’s qualifications, institutional affiliation, and the objectives of the study. The research was conducted in Jaén, a province in Andalusia, southern Spain. Initial contact was facilitated by a professional affiliated with the institution, and face-to-face interviews were scheduled. Importantly, none of the participants opted to withdraw from the study.

### 2.3. Methods and Data Collection Instruments

Data collection involved the use of semi-structured interviews [23,24] with a sample of 14 participants. The interviews were conducted by a skilled researcher (PhD student in Psychology, Social Worker; J-A-C-R), who has extensive experience in qualitative research and gender studies, particularly in the masculinities field.

The interview guide included questions designed to explore the influence of hegemonic masculinity on the addiction pathways of men undergoing treatment, particularly regarding sexuality issues (e.g., questions exploring the impact of addictions on sexuality, experiences with prostitution and pornography, and the influence of hegemonic masculinity and heteronormativity in their sexuality and addictions, among others). Furthermore, participants were invited to share their perspectives in depth on the topic and suggest new categories, which were later identified as emerging themes.

The interview guide, consisting of 12 questions, was first piloted with 10 male residents from the therapeutic community of Project Male Jaén. To enhance the study’s credibility [24], the guide was revised based on feedback from the pilot phase, as well as recommendations from two esteemed professors at the University of Murcia with expertise in qualitative research and gender studies. During the pilot interviews, valuable feedback was gathered, particularly regarding the technical language of some questions. To improve clarity, the questions were revised for better understanding. Participants also provided input at the end of the interviews, offering their perspectives on the questions and suggesting changes to ensure the study’s objectives were met. The main adjustments involved replacing scientific terms with language more accessible to the participants. Additionally, the pilot study confirmed that the interview was conducted within the planned time frame and aligned with the study’s objectives.

Each interview, held in a private and confidential space within the institution, lasted approximately one hour. It is important to note that no follow-up interviews were conducted. In addition to the information obtained through the interviews, participant observation methods [25] were also utilized to enrich the data collected. The observation enabled the analysis of non-verbal information during the interview and its comparison with the verbal data obtained. The lead interviewer recorded all relevant categories in the field diary. These categories were coded and included the following aspects: facial expressions, body language, gestures, and vocal qualities related to pauses and tone. Furthermore, within each of these categories, the following indicators were observed: occurrence, sequence, frequency, duration, intensity, and functionality.

The semi-structured interview approach follows a predefined framework covering the key topics to be addressed, with each session lasting around one hour. Throughout the process, strict confidentiality protocols were upheld. As noted earlier, semi-structured interviews are characterized by a structured guide that helps explore predefined topics. However, this format also allows participants the flexibility to share personal stories or insights beyond the outlined questions, thereby enriching the data and deepening the understanding of their perspectives [26].

### 2.4. Data Processing

Before and after each interview session, detailed field notes were carefully recorded. Additionally, with the participants’ explicit consent, audio recordings were utilized to ensure accurate data collection. Each transcription was anonymized by assigning a unique identification code (designated as “I of interviewee and the number of participant”), ensuring the confidentiality of the participants. The transcribed data utilized a specific orthographic convention to accurately document non-verbal cues and nuanced details, in accordance with established guidelines [27,28].

### 2.5. Data Analysis

This study employed a content analysis methodology [23,29], specifically utilizing conventional content analysis as outlined by Hsieh and Shannon [30]. This method involves generating codes directly from the data, which aids in the formation of categories. A significant advantage of this approach is its adaptability, allowing for the inclusion of new categories as they arise. The coding was conducted by authors J.A.C-R., C.M.G-S., and F.G., with ATLAS.ti software, version 22 (Scientific Software Development GmbH, Berlin, Germany) used for coding and subsequent analysis, and revised by the senior author R.M.L-G.

**Figure 1:**
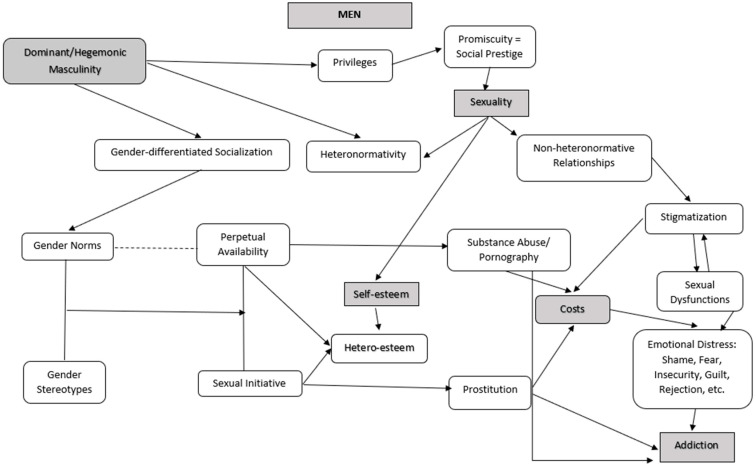
Interconnection among hegemonic masculinity patterns, addictions, and sexuality categories in men.

The content analysis process involved a comprehensive review of each transcription, where the text was segmented according to the research objectives. Codes were identified through an inductive analysis approach. These codes were then organized into categories using a deductive method. Inductive reasoning involves deriving general conclusions from specific observations. This approach was primarily used when emerging categories were identified from the verbatim transcripts and/or observations. In contrast, deductive reasoning is based on general principles to draw specific conclusions. This type of reasoning was primarily used to analyze the pre-existing categories, which are grounded in the existing literature [23,29,30]. Categories were classified as either a priori, based on existing literature, or emergent, derived from participants’ insights. Each category was defined, and emergent subcategories were conceptually outlined. Quotations from participants were used to support all elements [23,29]. Figure 1 presents the category tree, illustrating the main relationships between various themes and subthemes.

The content analysis was conducted through semantic, syntactic, and pragmatic lenses. Semantically, it involved examining the structure of the text and identifying specific lexical items, noting their sequence and evaluative meanings. Syntactically, the analysis compared the linguistic expressions of interviewees with established categories to identify both similarities and differences. From a pragmatic perspective, the focus was on contextual subtleties at both micro and macro levels, as well as the communication circumstances and their influence on the application of analytical categories [23,29].

Furthermore, the study utilized a triangulation methodology in terms of both methods and sources [31]. Methodological triangulation involved employing several data collection techniques, including semi-structured interviews and participant observations. In addition, source triangulation was implemented, considering the perspectives of individuals with a wide range of different characteristics and life experiences [29].

### 2.6. Ethical Considerations

The research project received approval from the Ethics Committee of the University of Murcia (M10/2024/329). The study protocol and questionnaire were supplemented by an informed consent form and an information sheet outlining the research’s aims and methodology. Participants were clearly notified of their right to withdraw from the study at any time if they wished to discontinue their involvement. Furthermore, consent was obtained from participants for the use of their data, thereby upholding the bioethical principle of autonomy.

Participants were guaranteed adherence to the principles of beneficence and non-maleficence, as the research sought to avoid causing harm while aiming to deepen the understanding of their situations. Additionally, the results obtained were shared with the institution to help enhance knowledge for improving care quality. Transparency was prioritized throughout the research process, with participants receiving feedback on the findings, and their contributions were accurately transcribed. All participants provided written consent, confirming their awareness of their right to withdraw from the study at any point. The study complies with relevant legislation, the Declaration of Helsinki, and established good clinical practice guidelines.

### 2.7. Strategies to Ensure the Quality of the Study

In alignment with Guba’s recommendations [24] for improving reliability, as well as the adherence to the Standards for Reporting Qualitative Research (SRQR) [19] and the Consolidated Criteria for Reporting Qualitative Studies (COREQ) [20], the following measures were implemented:

Credibility: To enhance credibility, both methodological and data source triangulations were utilized, along with detailed field notes. Moreover, participants were involved in the validation process by reviewing their transcripts and attending a group meeting to discuss the main findings. Additionally, external validation was obtained from two professors at the University of Murcia, who are acknowledged experts in qualitative analysis and gender studies.

Transferability: The segmentation and inclusion criteria were explicitly defined, and a thorough description of the data collection context was provided, offering valuable insights into both the process and the contextual subtleties involved.

Dependability: A detailed presentation of the theoretical framework and methodological design was provided, ensuring transparency and clarity in the research methodology.

Confirmability: Methodological and data source triangulations were reiterated to strengthen the confirmability of the findings. Additionally, the researchers critically examined their biases and stereotypes to minimize their potential impact on the coding process. Reflexive discussions were held with other qualitative researchers to assess and address possible biases. These discussions were documented and analyzed to prevent any future interference with the data analysis.

Furthermore, the accuracy of the results and transcripts was validated through participant involvement. After the interviews, participants convened with the researcher to review their pre-printed transcripts, providing corrections and confirming the accuracy of the presented information.

## 3. Results and Discussion

This qualitative research explores the complex relationship between male sexuality and addiction in men, with a particular emphasis on how constructs of hegemonic masculinity shape the sexual health of this population. The findings reveal that factors such as the pressure to maintain constant sexual availability, excessive engagement with prostitution and pornography, the emergence of sexual dysfunctions, and the reinforcement of values prioritizing sexual initiative are closely linked to substance abuse and the development of addictions. Additionally, the study highlights the role of challenges associated with oppression rooted in heteronormativity—understood as the imposition of rigid norms governing sexual orientation and behavior—in perpetuating these issues. This research seeks to deepen understanding of how these interconnected factors impact the physical and emotional well-being of men undergoing addiction treatment, emphasizing the need for more inclusive and reflective approaches in therapeutic practices.

In the following section, the key findings obtained from the analysis of the interviews are presented and discussed. The Results and Discussion section is organized as follows: (1) Sexuality and Addiction: Unraveling Men’s Experiences; (2) Pornography and Prostitution: Experiences of Men with Addictions; and (3) Sexuality and Heteronormativity Among Men with Addictions. Figure 1 offers a visual representation that aids in exploring the relationships among the several analyzed categories.

### 3.1. Sexuality and Addiction: Unraveling Men Experiences

Sexuality is an integral and complex dimension of human existence, where psychological, biological, social, and interpersonal factors intersect, each playing a fundamental role in its development and healthy functioning [32]. This multidimensional balance is inherently delicate, meaning that any factor—whether external or internal—that disrupts it, such as stress, relationship conflicts, sexual monotony, or biological imbalances, can result in dysfunctions in sexual response and satisfaction. Substance use can profoundly affect these domains, as it influences the physical, social, and psychological aspects of sexuality, leading to disruptions that frequently impact desire, performance, and sexual satisfaction. This multifaceted influence of drugs helps explain why substance abuse is commonly associated with significant negative effects on sexual health and overall well-being [33].

During adolescence and among young men, certain substances are commonly associated with sexual experiences. This practice, known as chemsex, entails the use of specific substances before or during organized sexual activities to enhance, initiate, prolong, sustain, or intensify the experience [34]. Chemsex has been identified as an emerging public health concern, particularly among men [34]. For example, alcohol remains the most commonly used substance and is perceived as offering the greatest benefits among the four sexual practices analyzed, including facilitating sexual encounters, engaging in riskier experiences, increasing arousal, and prolonging sexual activity [35,36]. However, when the goal is to extend the duration of sexual activity, cocaine tends to be the preferred substance [35]. Despite this, the use of these substances has significant negative consequences for sexual health, many of which participants remain unaware of. Some interviewees remarked:

*“When I was younger and began using substances, before having sexual relations with my partner, I preferred being high on cocaine because it helped me last longer in bed. I enjoyed it more, and my partner even more so”.*

(I 2)

*“When we were under the influence of alcohol, we (referring to their partner) engaged in sexual activities that we normally wouldn’t do. I always took control of the situation, and we also didn’t use any protection (contraceptives); we liked it better that way”.*

(I 7)

This perspective underscores two key aspects directly associated with the construction of hegemonic or dominant masculinity. The first relates to the use of drug consumption as a strategy to reinforce and perpetuate the concept of virility, defined as a display of power and dominance within the sexual sphere. In this context, substances are utilized to prolong sexual performance and enhance endurance, positioning men as providers of desire and reinforcing the norm of being active and dominant participants in sexual relationships. This perspective reflects the internalization of societal expectations that link masculinity to physical performance and the assumption of a predominant role in sexual encounters, thereby reinforcing stereotypes that sustain gender inequalities and power imbalances in relationships.

The second aspect is that, in the context of drug use, men may experience situations where drug consumption coincides with high-risk sexual behaviors, seeking to engage in impulsive, intense, and uncontrolled sexual encounters. In this way, substances serve as conduits to sexual experiences that deviate from the norm and lack safety [9].

Generally, it has been observed that the use of substances such as alcohol, benzodiazepines, cannabis, cocaine, opioids, amphetamines, methamphetamines, and hallucinogens, in low doses and over short periods, can lead to temporary improvements in certain aspects of sexual functioning [33]. These effects may include increased disinhibition, reduced anxiety, or heightened pleasure, which can lead to the belief that such substances enhance sexual performance. However, these improvements are temporary, as the body quickly develops tolerance, reducing their initial effectiveness. When use persists or doses increase, adverse consequences begin to prevail. Consequently, when consumed in higher doses or over extended periods, these substances can have significant negative effects on both the physiological and psychological aspects of sexuality [33].

In line with previous findings, some participants experience sexual dysfunction associated with their addiction and tend to avoid seeking help for this issue. Previous research has confirmed the negative impact of addiction on male sexuality [37,38] and the tendency of male populations with addiction to avoid seeking help, primarily due to the influence of hegemonic masculinity and related stigmas, such as appearing weak or insufficiently masculine [39]. It has been noted that, on many occasions, the act of maintaining initiative in sexual encounters is viewed as a cost of hegemonic or dominant masculinity [7]. This pressure often creates significant strain on men, as they are expected to consistently maintain sexual initiative. When combined with sexual dysfunction, this can lead to considerable distress for many men, which sometimes manifests as problematic substance use. This is also true for the stereotype that men are always expected to be willing to engage in sexual activity. Furthermore, many treatment centers fail to address sexuality or to effectively manage sexual dysfunctions related to substance use [37]. Participants explicitly expressed:

*“Although it was very difficult for me before because I always had to be available. If I couldn’t get an erection, I never talked about it; I never thought to discuss it with friends because I was afraid of what they might think of me”.*

(I 3)

*“I always believed that a man had to be available, ready for anything, especially sexually. When I started to fail, I felt less like a man. Alcohol helped at first, but then it only made everything worse”.*

(I 5)

*“On many occasions, when I had been using substances, I couldn’t maintain sexual activity, so I would buy Viagra illegally. To this day, I still have these issues, even though I don’t use drugs. I have never seen a specialist because I’m too embarrassed to talk about it”.*

(I 10)

*“I never spoke to anyone about my sexual problems because I was embarrassed. I felt that men cannot show weakness, especially in something as important as sex”.*

(I 13)

Previous research on alcohol-induced sexual dysfunction has primarily focused on issues such as erectile dysfunction and premature ejaculation, confirming the negative impact of alcohol on sexuality [40]. The relationship between drug use and sexual dysfunction involves a complex interplay of biological, psychological, and sociocultural factors, warranting further investigation.

Indeed, the connection between sexuality and addiction is multifaceted, and it is crucial that men are informed about this relationship to better manage the potential side effects during their rehabilitation. In this context, an improvement in sexual response is often observed simply by discontinuing substance use. However, another important consideration is that premature ejaculation, which affects 10.41% of men during periods of substance use, increases to 45.83% after substance cessation. At first glance, this statistic may seem contradictory. One possible explanation is that, during the initial sexual encounters after discontinuing substance use, men report heightened arousal for several reasons. First, they engage in sexual activity without any “intermediary”; in other words, there are no substances in their system to mask sensations or emotions, allowing them to feel “clear-headed.” Furthermore, the time elapsed since their last sexual encounter, often due to the detoxification and rehabilitation process, may contribute to this heightened state of arousal. Another possible explanation is the rebound effect on sexual response caused by the absence of substances. Del Río et al. [41] point out that cocaine use delays ejaculation and orgasm, prolonging sexual activity beyond its typical duration. Therefore, once the substance is eliminated, individuals who have used cocaine may experience a rebound effect, resulting in rapid ejaculation during sexual activity. Consequently, when addressing the issue of sexuality among men undergoing substance use cessation, it is essential to approach the topic of premature ejaculation not as a problem, but as a natural part of their recovery process.

Another important issue to consider when analyzing sexuality and addiction is the expectation of constant sexual availability among men, a key component of hegemonic masculinity. It is often assumed that men are always ready for sexual activity [1]. The idea that a man might not want to engage in sexual activity when a woman initiates it seems unusual, or even offensive [1]. When asked whether men should always be available for sex and what occurs when they do not feel inclined, the responses were as follows:

*“Yes, I have done it, and I did so to please my partner. When I was unable to engage in sexual activity, I felt humiliated and ashamed”.*

(I 2)

*“Although it was very difficult for me at first because I always had to be available. If I couldn’t achieve an erection, I didn’t talk about it; I never considered discussing it with friends, as I feared what they might think of me”.*

(I 3)

*“Once, it happened, and my wife thought I was being unfaithful, so I felt compelled to have sex. If she asks me to have sex, I have to comply”.*

(I 4)

*“When I told my girlfriend that I wasn’t in the mood, she felt awkward, and the same thing happened with a friend. I felt rejected”.*

(I 7)

*“Yes, I have always believed that a woman must be satisfied. The man must always be available. I am afraid of not measuring up to the expectations of a man”.*

(I 8)

*“Yes, this has happened to me. In fact, I’ve felt that I had to maintain a facade, that I had to meet women’s expectations. I have to be the ‘alpha male’”.*

(I 12)

Consequently, sexuality is directly linked to self-esteem and the challenges participants have faced in relation to their addiction. The participants explicitly expressed:

*“I always felt that I had to prove I could handle everything, even in bed. If I failed there, I felt like I was worthless as a man. This definitely increased my cocaine use, to last longer, but then I couldn’t stop, and that just dragged me down even more”.*

(I 3)

*“Using substances was a way to forget that I wasn’t living up to what was expected of me as a man in sex. I thought I was worthless because I also lost my job. It was a vicious circle: I felt less, used to escape from thinking, but in the end, it made me feel worse about myself”.*

(I 5)

*“I never realized how much sex affected my self-esteem until I had to talk about these issues here at the center. I always thought that if I wasn’t sexually active as a man, I wasn’t good enough to be with a woman”.*

(I 12)

In fact, it may be more appropriate to refer to this concept as “hetero-esteem” [42], particularly in relation to the reinforcement of masculinity within peer groups. This notion aligns with other gender norms promoted by hegemonic or dominant masculinity regarding the definition of manhood, including being the primary breadwinner and the authority figure within the family. These gender norms are also interconnected with others across various aspects of men’s lives, including their sexuality and experiences of failure in this domain. Analyzing these norms can deepen our understanding of the significant role that hegemonic masculinity plays in the addiction processes of many men.

In this regard, gender norms dictate that a man “must” always be willing and prepared to engage in sexual activity, reinforcing the idea that virility is directly associated with the frequency or “quantity” and “quality” of his sexual performance. This pressure perpetuates the belief that any failure in this area not only affects self-esteem but also calls into question his masculine identity, often linking it to stigmas such as the fear of being perceived as homosexual. As a result, male sexuality becomes a space of constant scrutiny and validation, where failure to meet these expectations is seen as a direct threat to personal worth. This framework helps us understand how negative sexual experiences, difficulties in meeting these expectations, and perceived failures in other aspects of life contribute to the development of addiction in many men. Substance dependence may emerge or worsen as a way of coping with the anxiety, shame, and sense of inadequacy stemming from these gender norms.

At the same time, the majority of participants interviewed expressed negative reactions toward women when they themselves did not want to engage in sexual activity. At times, they displayed anger, while in other instances, they portrayed themselves as victims, blaming the woman and making her feel as though she controlled the sexual dynamic, allowing it only when it suited her. This shifts the responsibility for sexual activity onto women, which, for some of the men interviewed, has been used to justify seeking sexual encounters outside the relationship. There is a pervasive societal belief that men are obligated to engage in sexual activity [1] and also enhance promiscuity in men as a positive value [7]. Participants specifically highlighted:

*“I don’t like it when she says that; she can always engage in sexual activity, but I can’t. I react immaturely and become angry”.*

(I 1)

*“Honestly, I’ve felt quite angry. I experienced frustration and powerlessness, which made me feel upset”.*

(I 4)

*“I stopped communicating with her. I became angry and expressed that she was not considering my feelings”.*

(I 6)

*“Recently, I felt upset because I thought she no longer liked me or that she might be seeing someone else. I felt worthless”.*

(I 11)

*“I became angry, to be honest. I kept insisting until I could achieve my desired outcome”.*

(I 14)

For men with addiction issues, the system of privileges associated with promiscuity is not without its costs. Hegemonic masculinity demands that men meet unattainable standards of virility [1], which often drives them to use substance use as a tool to maintain a constant state of sexual availability and ensure their “success” in the sexual domain. Another cost, according to Lozoya [7], is that men are expected to display calmness and rationality in the face of sexual rejection. However, this is not always the case, as there exists a belief that men must succeed in their sexual advances, leading to situations that often generate frustration and anger. As indicated in the interviews, this sometimes results in conflicts with their romantic partners. Nevertheless, these practices often lead to negative consequences, such as emotional dependence on external validation, the deterioration of affective relationships, which complicates the establishment of authentic and healthy connections, and, in many cases, results in substance abuse, leading to both emotional and physical costs.

Moreover, half of the participants hold a very negative view of women who freely engage in sexual relationships whenever they desire, particularly outside of committed partnerships. Some men perceive this positively, as it enables them to have more sexual encounters, rather than stemming from any notion of freedom for women. Participants notably mentioned:

*“I believe she enjoys being with everyone, and in the end, she won’t be able to settle down with anyone”.*

(I 1)

*“Well, I think she isn’t thinking clearly and doesn’t consider the implications of her actions”.*

(I 3)

*“In terms of sexual freedom, this allows me to have more sexual relationships”.*

(I 2)

*“I think it’s acceptable, except when it’s with a girl I like; then it annoys me”.*

(I 5)

*“She is promiscuous”.*

(I 6)

*“A woman like that is worthless; she’s just anyone”.*

(I 8)

These dynamics result from a gender-differentiated socialization that perpetuates inequalities between men and women, particularly in the realm of sexuality [15]. In this context, a moralistic view of sexuality has been consolidated, granting symbolic and social privileges to men, while for women, it serves as a source of oppression and social rejection. Male promiscuity, for instance, is often valued and associated with prestige and status within peer groups, thereby reinforcing their masculine identity and solidifying their position within patriarchal power structures. In contrast, women who engage in similar behaviors face significant social stigma, which underscores the asymmetry in the valuation of sexual behaviors based on gender.

On the other hand, the majority of the interviewed individuals reported negative experiences related to jealousy, stemming from a sense of possession toward their partners and influencing fears of sexual infidelity. They position sex as the culmination of passionate love, with exclusivity serving as a primary norm. Participants notably mentioned:

*“I also became jealous of my partner when I drank, and then I became possessive”.*

(I 4)

*“I have experienced negative feelings in all three instances, especially regarding possession”.*

(I 5)

*“I have had issues with jealousy in my romantic relationships, trying to control my partner sexually”.*

(I 6)

*“I was a possessive and controlling person, and I even resorted to abusive behavior because of it”.*

(I 8)

*“I also faced jealousy issues with my partner because the relationship was solely based on sexual interactions. She liked it when I dominated the relationship”.*

(I 11)

This notion also reflects the myths of romantic love [43] such as jealousy and possessiveness, which, in turn, undermine intimate relationships and have been shown to be associated with emotional dependency and gender-based violence, among other issues [44,45]. Furthermore, the myths of romantic love are reinforced by the patriarchal system, which utilizes them as mechanisms to sustain the balance of power in favor of men and to bolster the mandates of hegemonic masculinity [45].

As previously stated within the framework of hegemonic masculinity, jealousy assumes a symbolic role that not only reinforces male dominance but also serves as a mechanism to ensure control over the partner. This control is directly tied to the validation of male virility and status within his social environment [15]. The link between jealousy, the myths of romantic love, and addiction issues in men lies in how these social constructs, in some cases, intensify the emotional and psychological pressure of maintaining constant control over another person. On the one hand, jealousy heightens the need to demonstrate power and authority within the relationship, potentially leading to behaviors that cause problems in romantic relationships.

This scenario is further exacerbated when considering the influence of gender-differentiated socialization, which not only encourages possessiveness and control as desirable traits in men but also reinforces the perception that displaying vulnerability, insecurity, or a need for emotional support is a sign of weakness. As a result, men often struggle to recognize and manage their emotions effectively, leading them to seek relief through substance use as a means of escaping their internal conflicts and avoiding help-seeking behavior [39].

### 3.2. Pornography and Prostitution: Experiences of Men with Addictions

The results of this study indicate a high consumption of pornography among the participants. Along with peer groups, it has been the primary source of information about sexuality for the men involved in the study. In fact, thirteen out of the fourteen interviewed individuals (92.86%) do not hold a negative view of pornography; quite the opposite. Although this topic is discussed only among men and never with women, the participants perceive discussing pornography with women as inappropriate. Participants explicitly noted:

*“I like it because I enjoy sex, and I have it readily available; I also don’t see it as something harmful to anyone; it is a source of pleasure for me”.*

(I 1)

*“I like using it because it motivates me for sexual activity and excites me. It also serves as a learning tool, and sometimes I put into practice what I see; I appreciate it for that”.*

(I 3)

*“I like it; it is a service and very commonplace; it’s there to be used. It allows me not to have to pursue a woman; with pornography, it’s easier. When I was younger, it was forbidden, and I sought it out; for me, having access to so much pornography has been liberating”.*

(I 7)

It is also noted among the interviewees that pornography is often associated with substance use. Pornography consumption can influence sexual behaviors, contribute to sexual dysfunction, and negatively shape attitudes toward sexuality [46]. Participants notably mentioned:

*“If pornography didn’t exist, sex would be worse. ’I’m addicted to it. I like to watch it, especially when I’m quite intoxicated”.*

(I 2)

*“In fact, the most pornography I have ever watched was when I was using substances”.*

(I 3)

*“I think I have a serious problem with pornography. Since being at the center, I have talked about this in group several times. When I think about the times I watched pornography while using substances, I feel guilty. I have had problems with some partners because of this”.*

(I 5)

*“Whenever I stayed home using substances alone, I did it while watching pornography”.*

(I 7)

*“It’s something simple to watch and enjoy. I have consumed it while using cocaine”.*

(I 10)

*“I have consumed it daily, even while being with a partner. It has caused me discomfort many times because I used it so much; I think I became addicted”.*

(I 13)

Pornography generates addictive behaviors that can sometimes develop into comorbidity issues among individuals with substance addictions. This phenomenon unfolds in three distinct phases. First, a process of overstimulation occurs when viewing behaviors activate the body’s reward system, resulting in increased levels of dopamine, a neurotransmitter associated with pleasure. Next, withdrawal syndrome manifests due to the overstimulation of dopamine receptors, which require higher amounts of the neurotransmitter to achieve the same pleasurable effect. This overstimulation impacts the systems responsible for stress and anxiety, prompting individuals to seek additional pleasurable stimuli to alleviate unpleasant sensations. Finally, the stage of concern emerges, wherein neural plasticity altered by consumption affects areas related to self-control [47].

Viewing explicit sexual content increases dopamine release in the brain, particularly affecting the frontal lobe, which regulates behavioral and cognitive functions. In summary, consuming pornography has effects analogous to those of substances such as cocaine, leading to addictive behaviors characterized by hyperreactivity to stimuli, diminished pleasure, and loss of control [47]. Furthermore, these addictive behaviors can result in physical, psychological, emotional, and spiritual problems for the individual [48].

Moreover, pornography appears to function as a means of seeking a more satisfying sexual life outside of current relationships and/or alleviating feelings of loneliness. This, in turn, suggests the need for enhanced sexual and emotional education for individuals with addictions, thereby providing them with more resources to cope with loneliness and address sexual frustrations in a healthier manner. It would also be beneficial for them to establish more effective communication with their partners in order to resolve sexual problems or dissatisfaction through alternative approaches. The use of pornography as a coping mechanism for loneliness not only reflects an individual strategy for managing emotional distress but also underscores a greater adherence to gender norms associated with hegemonic masculinity. This practice reinforces the emotional and social costs linked to this model of masculinity, as many men, conditioned by these norms, tend to avoid seeking emotional support or displaying vulnerability. Rather than confronting their loneliness through meaningful interpersonal connections, they opt for individualistic and depersonalized solutions, thereby perpetuating dynamics of isolation and reinforcing an image of self-sufficiency that, in the long term, may exacerbate their emotional distress and social disconnection [1,39]. Some participants indicate:

*“It’s something simple to watch and enjoy when you’re not satisfied with your sex life. I used it daily and would watch videos to enjoy them”.*

(I 10)

*“I have used it extensively, and I feel guilty afterward, particularly when I consume it to satisfy myself due to feelings of loneliness”.*

(I 12)

Moreover, some of the men express a contradiction regarding pornography. Despite their high level of consumption, they often articulate that using it causes them discomfort. In fact, some of the interviewed men believe that certain videos are degrading to women, yet they consume pornography daily. Although it is observed in only a few cases, it reveals instances of gender awareness concerning the subordinate position of women in pornography and its consumption as part of the patriarchal model within which hegemonic masculinity is situated. Participants particularly emphasized:

*“It degrades women because it depicts actions that portray them negatively, such as spitting on and hitting them”.*

(I 11)

*“The way women are portrayed in some of the videos is outrageous; the treatment of women is appalling”.*

(I 10)

*“Yes, it degrades women; women are treated as objects in the material I have consumed”.*

(I 12)

In addition, prostitution has been engaged in by eleven of the fourteen individuals interviewed (78.57%). The majority of the men associate it with addiction, indicating that they have utilized prostitution concurrently with drug use. For these individuals, there is a perceived connection between drug use and the initiation of prostitution. In most cases, they have frequented venues where prostitution occurs alongside their male peers. It reinforces the hegemonic masculinity in which a man is not stigmatized for engaging in prostitution [7]. Moreover, it is also confirmed that in some cases prostitution often functions as a rite of passage into heterosexual relationships, frequently reinforcing notions of virility through group dynamics [5]. Such initiations are often associated with excessive consumption of alcohol and other substances. In fact, some men recognize a relationship among prostitution and drug use, viewing drugs as way to be uninhibited that facilitate engagement in these activities. This reflection emerged during the interviews, as most participants did not perceive a connection.

*“Many times, because I was consumed and that made me morbid. When I have consumed prostitution, I have always consumed”.*

(I 1)

*“The consumption of prostitution combined with the use of other substances makes me feel guilty. Above all, I regret seeing all the money spent. I always associate prostitution with consumption. The consumption has disinhibited me to be able to use prostitution. Being with prostitutes has sometimes brought me many problems”.*

(I 2)

*“The consumption of prostitution has been negative for me, as I combined it with substance use. Now that I think about it, it was a way of exerting control through money, as this way I could do whatever I wanted”.*

(I 3)

*“Consumption and prostitution became a kind of escape. At first, it seemed like they helped me, but over time, they only made me feel worse”.*

(I 5)

*“I have had a crisis due to the use of prostitution, because of the position I find myself in. I have had some difficulties in sexuality. Although I don’t view it as dominance, I see it as abuse”.*

(I 6)

*“I have used it to vent and other times to have company”.*

(I 7)

*“When I played the machines, I was going to consume myself and then consume prostitution. To satisfy sexual desires that could not be fulfilled any other way. Instead of going home, I was going to engage in prostitution, I started when I was 1, I started with friends. Whenever I used lately I used prostitution”.*

(I 10)

*“Yes. As a form of pleasure in which I am the one in charge, due to emotional lack as well. I think that a man who engages in prostitution has low self-esteem”.*

(I 12)

Furthermore, the feeling of guilt re-emerges as a tool for victimization. It is also evident that the majority have recognized the inequalities in power dynamics, as the ability to pay enables them to fulfill their sexual desires. In some cases, money and drug use empower men to take the initiative, a mandate deeply ingrained in hegemonic masculinity [5]. Financial resources and drug abuse position them as active subjects, helping them confront emotions such as fear and shame, thereby allowing them to access sex without needing to establish more equitable relationships. Additionally, these behaviors related to the consumption of prostitution and pornography prompt reflection on the nature of the self-esteem that men possess.

### 3.3. Sexuality and Heteronormativity Among Men with Addiction

Based on previous findings, it is also essential to dedicate a section to heteronormativity. Among the men interviewed, ten out of fourteen (71.43%) expressed tolerance for sexual relationships between men. However, all participants, without exception, have made jokes about homosexuals. In response to the question, all participants assumed that discussions of sexual relationships between men pertained specifically to homosexuality. It is another piece of evidence of how patriarchy and hegemonic masculinity, through differentiated socialization, have developed mechanisms—most of which are cruel and inhumane—to exclude men who may develop feelings or desires for individuals of the same sex. One of the most prevalent and damaging mechanisms has been the joking and ridiculing of homosexual individuals. Participants specifically pointed out:

*“Yes, because I feel compelled to laugh if I do not identify with them; it has happened to me on several occasions when I have witnessed jokes about homosexuals”.*

(I 5)

*“Yes, in fact, I have experienced it directed at me numerous times”.*

(I 6)

*“I believe it is normal for such jokes to be made; when I participate in these jokes, I reaffirm my masculinity”.*

(I 7)

*“Since I do not perceive it as normal, I have occasionally laughed at those jokes. Anything that approaches femininity is regarded as negative”.*

(I 8)

This aligns with the notion that hegemonic or dominant masculinity, grounded in patriarchal paradigms, enforces a narrow understanding of sexuality that favors heterosexual behaviors while penalizing those that diverge from heteronormative expectations [8]. Nevertheless, only a minority of participants appears to recognize the negative impact of this gender-differentiated socialization on their perceptions of homosexuality. They state the following:

*“Yes, I believe this is due to the education we have received and the perception that it is an attack on our masculinity. Jokes are often made because it is commonly believed that a homosexual is closer to being a woman”.*

(I 2)

*“Yes. Such jokes are made to demonstrate one’s masculinity and to avoid appearing feminine, primarily out of fear”.*

(I 12)

The patriarchy and its imperative of heterosexuality are fundamentally connected to sexual potency, which is passed down from fathers to sons, thereby converting it into a form of symbolic and social capital. This manner of transmitting sexuality not only repressively rejects but also perpetuates a homophobic sentiment that marginalizes alternative expressions of sexuality [8].

Other participants maintain a superficial tolerance for homosexuality, asserting their tolerance while, in reality, they do not hold such views. They claim indifference but express discomfort at witnessing it or being associated with homosexual men. Participants highlighted in particular:

*“I view it as acceptable; I respect it, but I don’t want anyone to say anything to me. I find it repulsive to see two men kissing. I dislike being perceived as anything other than a man; that bothers me. I don’t want people to think I’m gay, as it would make me feel rejected”.*

(I 4)

*“It’s strange; although I acknowledge that it is sometimes regarded as normal, it has made me uncomfortable”.*

(I 12)

*“I have no objection to others living their lives as they choose, as long as it doesn’t affect me or involve comments directed at me”.*

(I 14)

Many of the interviewed men have engaged in sexual relationships with other men within the context of addiction, a phenomenon that is more common than it may seem. Their thoughts and emotions are reinforced by guilt and shame, as they convey negative messages to themselves regarding their sexual orientation. These messages have often led to a direct increase in drug use [49], exacerbated by the fear of being rejected by other men. This is why many men refrain from discussing their experiences of this nature, which could help normalize such encounters and have often been crucial factors in drug abuse or addiction-related issues. Participants explicitly expressed:

*“I would not like to feel attracted to men, and I dislike thinking about that. I have never discussed it, and it really bothers me”.*

(I 1)

*“I have felt confused; that curiosity and desire should not have awakened in me. I worry about what other men will think, which poses a problem in my life”.*

(I 5)

*“I don’t handle this well; I feel embarrassed talking about it, and I’m not sure how my peers will react if I bring it up here. Until today, I had never discussed it in this way. On some occasions, I have had sex with other men to get money to buy drugs”.*

(I 13)

In certain contexts of drug use, men may become involved in problematic or oppressive sexual relationships—such as prostitution, sexual dysfunctions, abuse, or sexual violence—or in interactions that transgress established heteronormative norms. These experiences often trigger existential and emotional crises that contribute to both substance abuse and the development of addiction [9,50,51].

In addition, the pressure to conform to heterosexual expectations can create a profound sense of alienation and dissatisfaction among those who do not identify as heterosexual. This pressure may lead to feelings of shame, guilt, and diminished self-esteem, particularly in environments where sexual diversity is neither accepted nor valued. The invisibilization and stigmatization of non-heterosexual identities can hinder the authentic exploration and expression of sexuality, negatively impacting the sexual and emotional health of individuals engaging in non-heteronormative behaviors. Furthermore, the rigidity of patriarchal paradigms surrounding sexuality can manifest in forms of discrimination and violence against those who challenge established norms.

### 3.4. Strengths, Limitations, Future Lines of Research, and Clinical Relevance

One of the main strengths of this study is its novelty and the inclusion of gender as an analytical category. Additionally, employing masculinity as an analytical framework in health-related topics is highly relevant and addresses an increasing demand in this area. One of the main limitations, however, is the inability to generalize or propose causal hypotheses due to the methodological design.

A significant challenge emerging from these findings for psychologists, healthcare professionals, and social workers is the need to design interventions that incorporate a gender perspective and are specifically aimed at addressing addiction in men. It is important to note that, in most cases, men are not considered a distinct group in gender-focused interventions [2,3]. When they are included, these interventions rarely incorporate elements that question the roles and expectations associated with masculinity. This omission can be explained by the prevalence of an androcentric normative framework, which has historically positioned the Western, middle-class, Christian, middle-aged, and white male as the model when intervening with other marginalized groups [2,3]. Therefore, it is crucial that psychological interventions, especially those related to addiction treatment, not only acknowledge the structural inequalities that affect men but also critically address the gender norms and societal mandates that shape addictive behaviors and emotional barriers.

Regarding future lines of research, it would be valuable to explore the impact of hegemonic masculinity on women with addiction problems and to further examine the relationship between substance addiction and addictions to pornography and sex, particularly with respect to prostitution consumption.

A key aspect of clinical practice with men experiencing addiction issues is the need for a specific and systematic approach to sexuality throughout the treatment and social reintegration process. This approach should not only address the biological and behavioral aspects associated with addiction but also consider the cultural, emotional, and relational constructs that influence these experiences.

It is important to conduct a more thorough analysis of intersectionality within this context. Crenshaw [52] defined intersectionality as the way in which individuals experience oppression or benefit from privilege based on their membership in multiple social categories. This perspective is crucial in the field of addiction, particularly when attempting to intervene with individuals currently experiencing these issues. Beyond gender, it is essential to consider other variables such as sex, ethnicity, place of origin, social class, educational level, and economic status. These factors are key to understanding how gender is shaped in individuals with addiction problems and often influence their sexuality.

When analyzing challenges related to addiction and sexuality, it is critical to account for social class. The experience of being a man in a working-class or impoverished neighborhood is vastly different from that of a man in a wealthy area. Likewise, addressing issues of intimacy and sexuality with a man whose culture or education is grounded in sexist and unequal practices will differ from engaging with a man from a more egalitarian background. Age also plays a significant role; discussing these issues with a 65-year-old man is not the same as discussing them with a 19-year-old. Additionally, it is important to consider men who do not conform to heteronormative sexuality. As such, the analysis of these variables should go beyond simply including age and place of birth, and should provide insights that contribute to the development of more diverse and inclusive intervention strategies to improve the sexuality of men with addiction issues [39].

It is also crucial to incorporate an intervention framework that integrates a gender perspective, recognizing how hegemonic masculinity norms can shape both the perception and experience of sexuality. Furthermore, this approach should be based on individualized care that considers the subjectivities and particularities of each patient, avoiding generalized methods that may overlook a diverse range of behaviors.

In the context of treatment and social reintegration, addressing sexuality not only enhances the quality of life for patients but also supports a more holistic recovery, fostering greater emotional and relational autonomy, which in turn reduces the risk of relapse. This comprehensive approach therefore requires an interdisciplinary commitment that integrates therapeutic tools tailored to both individual and collective needs, while encouraging critical reflection on the power dynamics and inequalities inherent in gender relations—dynamics that have, at various points, contributed to sexual issues experienced by many men in treatment.

It is also important for social and healthcare professionals to receive training on gender issues and sexuality in order to effectively intervene in the field of addictions. Such training can also contribute to ensuring that individuals undergoing rehabilitation receive improved sexual education and develop greater emotional intelligence. In addition, sex education plays a fundamental role in preventing the consumption of pornography, prostitution, and drug abuse, as it equips individuals with the necessary tools to understand and critically evaluate the social norms and expectations related to sexuality. Comprehensive sex education not only addresses anatomy and physiology but also promotes the development of essential skills, such as informed decision-making and effective communication in relationships. By fostering a healthy understanding of sexuality and destigmatizing topics such as sexual desire and addiction, vulnerability to exploitation and risk behaviors can be mitigated. Furthermore, appropriate sex education can contribute to the establishment of healthier interpersonal relationships, where consent is valued and emotions are acknowledged, thereby reducing the tendency to seek escape through pornography, prostitution, and psychoactive substances as means of coping with emotional or social challenges.

## 4. Conclusions

In summary, after analyzing certain characteristics of patriarchal society and its socialization processes—insightfully highlighted by feminism over the decades—and based on the previous findings, it is observed that hegemonic masculinity significantly influences drug abuse and addiction problems among men.

It is important to emphasize the relationship between pornography consumption and substance addiction. Both activities are linked to behaviors associated with masculinity and are often learned within peer groups. Substance use reaffirms male courage through the risk involved in experimentation, while pornography reinforces the image of the virile, heterosexual man within the peer group. As previously noted, virility and courage are central values of hegemonic masculinity, instilled in men through gender norms and stereotypes shaped by socialization.

Gender-differentiated socialization and education have established mandates that regulate how men and women should experience and express their sexuality. These deeply ingrained gender norms form the foundation of interpersonal relationships and significantly influence early sexual experiences. In particular, peer pressure plays a crucial role in reinforcing behaviors that support these gender expectations, shaping attitudes and decisions related to sexuality, especially in adherence to dominant heteronormativity. Sexual encounters outside of heteronormative behaviors are sometimes linked to substance abuse.

The analysis of the interviews revealed the challenges many men face in trying to meet the demands imposed by hegemonic masculinity in the sexual domain. These tensions are reflected in issues related to problematic engagement with prostitution and pornography, practices that not only align with the expectations of dominance and objectification associated with the male role but also correlate with substance use and comorbidity dynamics. Pressures linked to gender mandates, such as the obligation to take initiative in sexual encounters, maintain constant sexual availability, and the stigma surrounding sexual dysfunctions, were also evident, often interpreted as an inability to meet virility standards.

In this context, it is essential to examine how these social and cultural constructions shape the experience of male sexuality, creating dynamics that not only affect the quality of interpersonal relationships but also have a significant impact on men’s emotional and psychological health.

This study underscores the importance of examining sexuality in men with addictions from a gender perspective, as well as the need for social and healthcare professionals to receive training on gender issues and sexuality in order to intervene effectively in the field of addictions.

Regarding future research directions, it would be valuable to explore the impact of hegemonic masculinity on women with addiction issues. Furthermore, further investigation into the relationship between substance addiction and compulsive behaviors such as pornography and sex addiction, particularly in relation to the consumption of prostitution, would be beneficial.

## Figures and Tables

**Table 1:** Participant characteristics.

Participant (P) Code	Age	Gender	Marital Status	Education Level	Profession	Months in Rehabilitation	Emotions Most Difficult to Recognize (Related to Sexuality)
**P1**	27	Male	Single	Primary School	Carpenter	7	Fear and shame
**P2**	41	Male	Single	University	Chemist	9	Anger, sadness, and pain
**P3**	49	Male	Married	Primary School	Truck Driver	6	Insecurity and joy
**P4**	34	Male	Divorced	Primary School	Welder	10	Fear
**P5**	29	Male	Single	University	Social Worker	7	Fear, pain, and guilt
**P6**	26	Non-binary	Common-law partner	Primary School	Caretaker	10	Fear
**P7**	37	Male	Single	High School Graduate	Hotelier	8	Love and shame
**P8**	25	Male	Divorced	High School Graduate	Pesticide Applicator	10	Fear
**P9**	50	Male	Married	University	Engineer	8	Fear, anger, and loneliness
**P10**	37	Male	Single	FP/Grado Medio	Maintenance Technician	9	Insecurity and shame
**P11**	28	Male	Single	High School Graduate	Maintenance Technician	7	Fear and joy
**P12**	51	Male	Divorced	FP/Grado Superior	Music Teacher	11	Fear, distress, and anger
**P13**	33	Male	Single	High School Graduate	Hotelier	10	Fear and guilt
**P14**	24	Male	Single	Primary School	Farmer	8	Fear and insecurity

## Data Availability

The data that support the findings of this study are available on request from the corresponding author(s).
